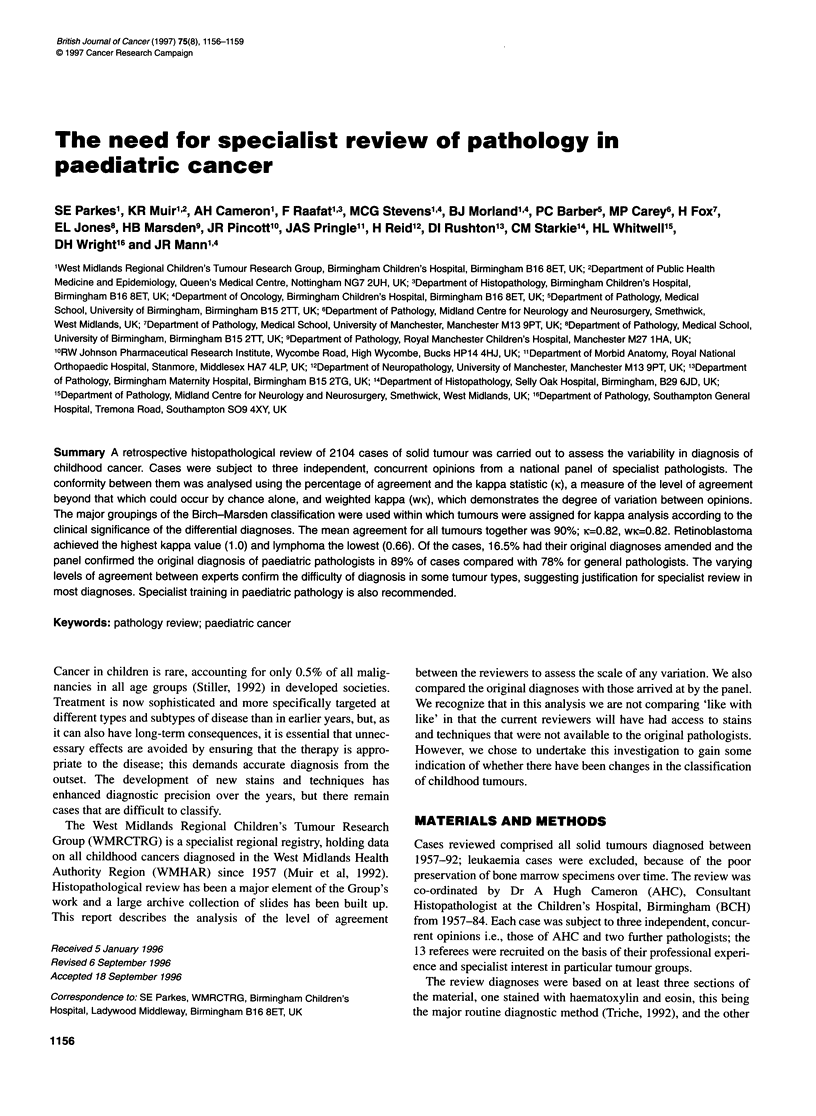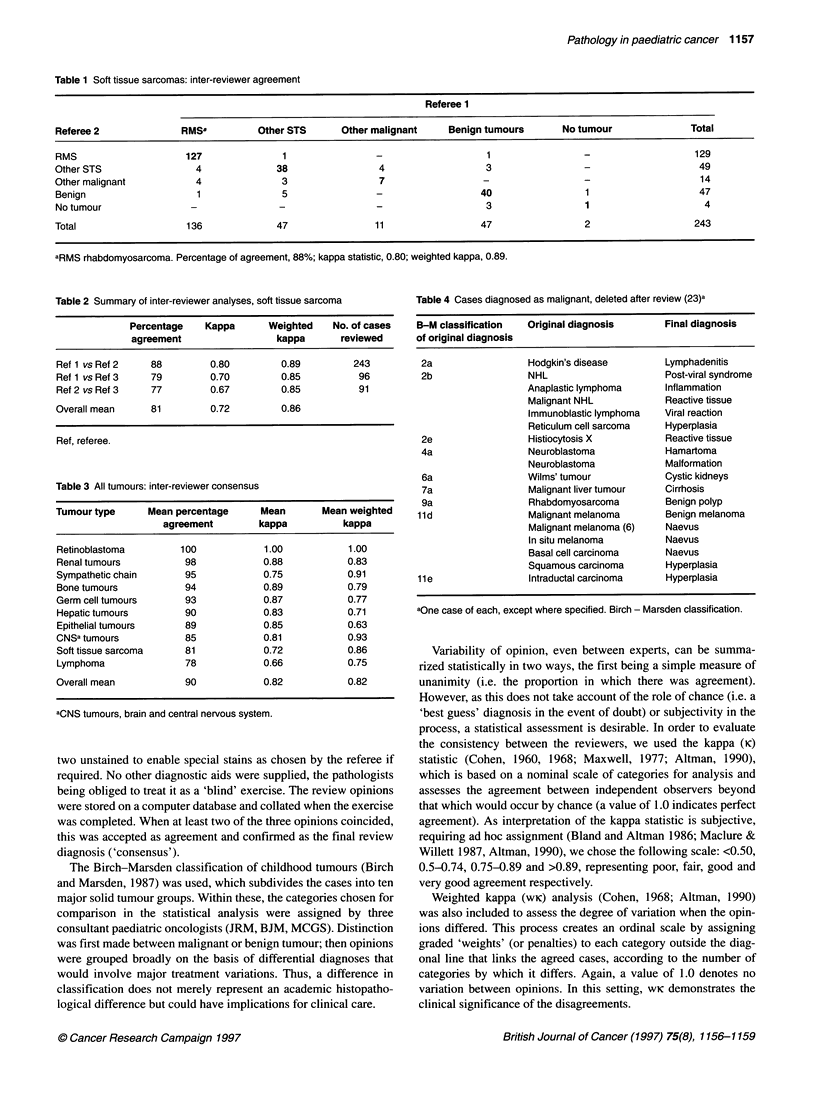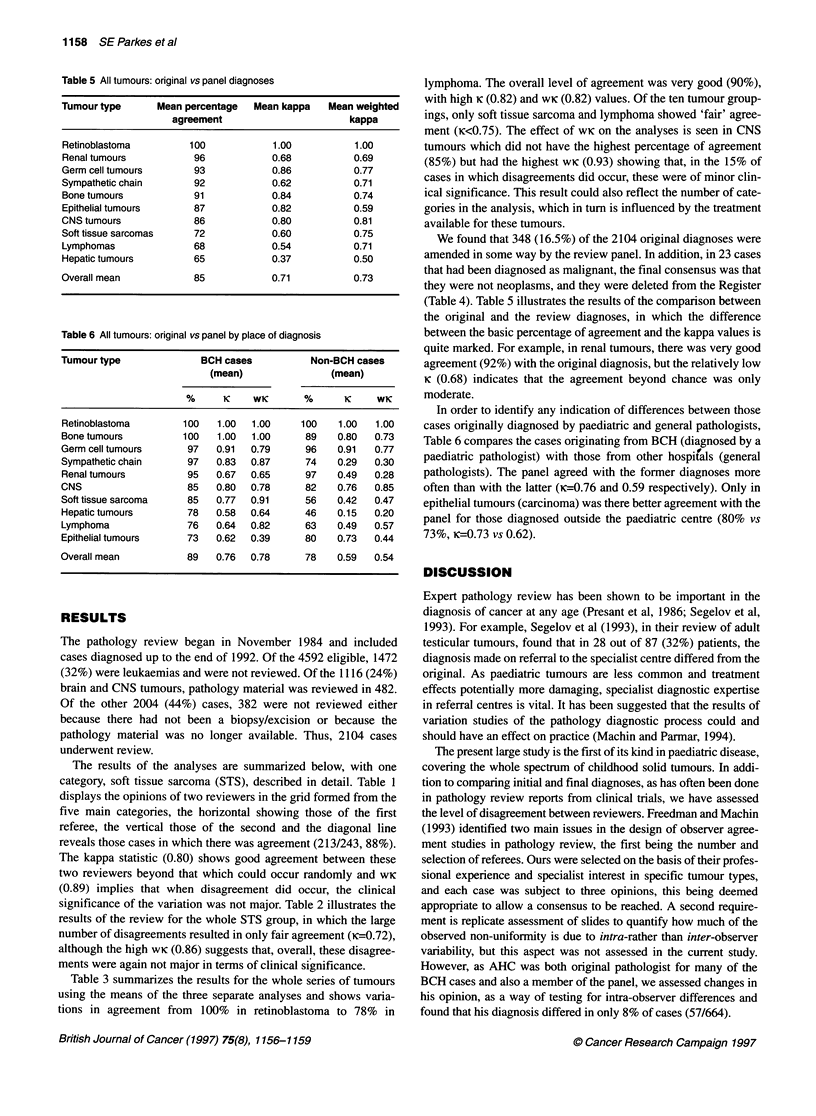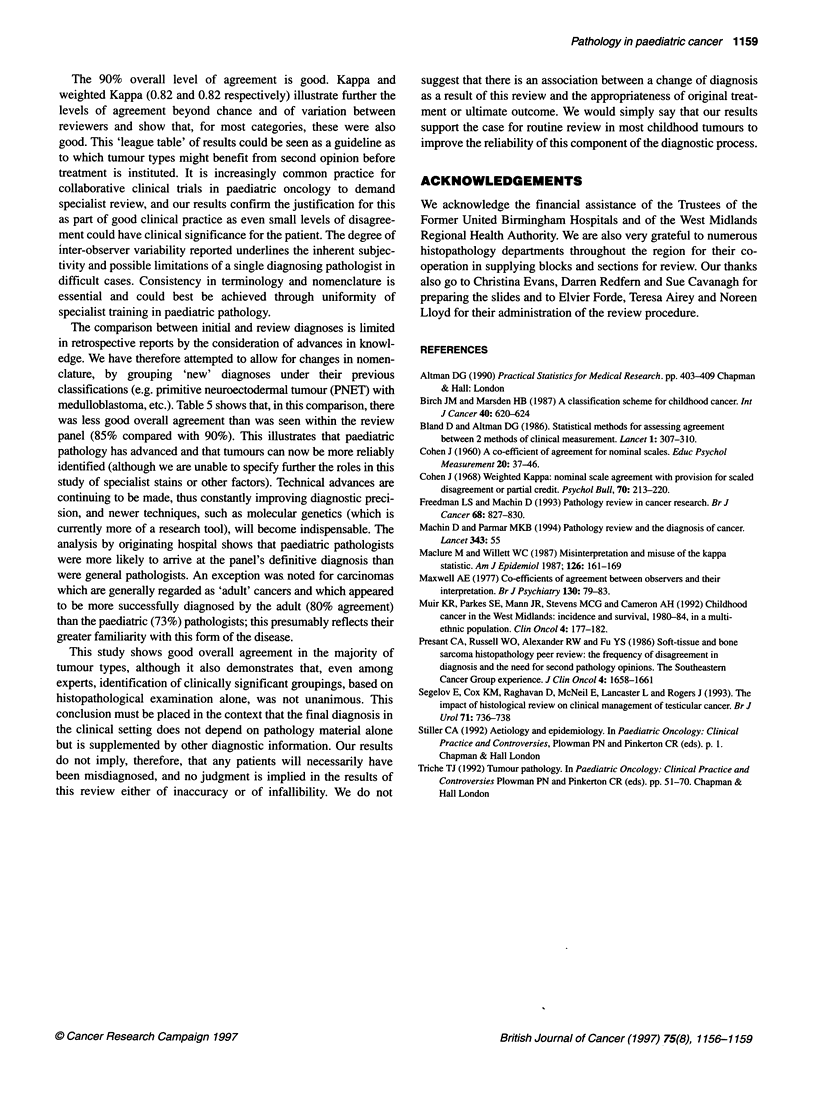# The need for specialist review of pathology in paediatric cancer.

**DOI:** 10.1038/bjc.1997.199

**Published:** 1997

**Authors:** S. E. Parkes, K. R. Muir, A. H. Cameron, F. Raafat, M. C. Stevens, B. J. Morland, P. C. Barber, M. P. Carey, H. Fox, E. L. Jones, H. B. Marsden, J. R. Pincott, J. A. Pringle, H. Reid, D. I. Rushton, C. M. Starkie, H. L. Whitwell, D. H. Wright, J. R. Mann

**Affiliations:** West Midlands Regional Children's Tumour Research Group, Birmingham Children's Hospital, UK.

## Abstract

A retrospective histopathological review of 2104 cases of solid tumour was carried out to assess the variability in diagnosis of childhood cancer. Cases were subject to three independent, concurrent opinions from a national panel of specialist pathologists. The conformity between them was analysed using the percentage of agreement and the kappa statistic (kappa), a measure of the level of agreement beyond that which could occur by chance alone, and weighted kappa (w kappa), which demonstrates the degree of variation between opinions. The major groupings of the Birch-Marsden classification were used within which tumours were assigned for kappa analysis according to the clinical significance of the differential diagnoses. The mean agreement for all tumours together was 90%; kappa = 0.82, w kappa = 0.82. Retinoblastoma achieved the highest kappa value (1.0) and lymphoma the lowest (0.66). Of the cases, 16.5% had their original diagnoses amended and the panel confirmed the original diagnosis of paediatric pathologists in 89% of cases compared with 78% for general pathologists. The varying levels of agreement between experts confirm the difficulty of diagnosis in some tumour types, suggesting justification for specialist review in most diagnoses. Specialist training in paediatric pathology is also recommended.


British Journal of Cancer (1997) 75(8), 1156–1159
© 1997 Cancer Research Campaign

### The need for specialist review of pathology in paediatric cancer

**SE Parkes[1], KR Muir[1,2], AH Cameron[1], F Raafat[1,3], MCG Stevens[1,4], BJ Morland[1,4], PC Barber[5], MP Carey[6], H Fox[7], EL Jones[8], HB Marsden[9], JR Pincott[10], JAS Pringle[11], H Reid[12], DI Rushton[13], CM Starkie[14], HL Whitwell[15], DH Wright[16] and JR Mann[1,4]**

[1]West Midlands Regional Children's Tumour Research Group, Birmingham Children's Hospital, Birmingham B16 8ET, UK; [2]Department of Public Health Medicine and Epidemiology, Queen's Medical Centre, Nottingham NG7 2UH, UK; [3]Department of Histopathology, Birmingham Children's Hospital, Birmingham B16 8ET, UK; [4]Department of Oncology, Birmingham Children's Hospital, Birmingham B16 8ET, UK; [5]Department of Pathology, Medical School, University of Birmingham, Birmingham B15 2TT, UK; [6]Department of Pathology, Midland Centre for Neurology and Neurosurgery, Smethwick, West Midlands, UK; [7]Department of Pathology, Medical School, University of Manchester, Manchester M13 9PT, UK; [8]Department of Pathology, Medical School, University of Birmingham, Birmingham B15 2TT, UK; [9]Department of Pathology, Royal Manchester Children's Hospital, Manchester M27 1HA, UK; [10]RW Johnson Pharmaceutical Research Institute, Wycombe Road, High Wycombe, Bucks HP14 4HJ, UK; [11]Department of Morbid Anatomy, Royal National Orthopaedic Hospital, Stanmore, Middlesex HA7 4LP, UK; [12]Department of Neuropathology, University of Manchester, Manchester M13 9PT, UK; [13]Department of Pathology, Birmingham Maternity Hospital, Birmingham B15 2TG, UK; [14]Department of Histopathology, Selly Oak Hospital, Birmingham, B29 6JD, UK; [15]Department of Pathology, Midland Centre for Neurology and Neurosurgery, Smethwick, West Midlands, UK; [16]Department of Pathology, Southampton General Hospital, Tremona Road, Southampton SO9 4XY, UK

**Summary** A retrospective histopathological review of 2104 cases of solid tumour was carried out to assess the variability in diagnosis of childhood cancer. Cases were subject to three independent, concurrent opinions from a national panel of specialist pathologists. The conformity between them was analysed using the percentage of agreement and the kappa statistic ($\kappa$), a measure of the level of agreement beyond that which could occur by chance alone, and weighted kappa (w$\kappa$), which demonstrates the degree of variation between opinions. The major groupings of the Birch–Marsden classification were used within which tumours were assigned for kappa analysis according to the clinical significance of the differential diagnoses. The mean agreement for all tumours together was 90%; $\kappa$=0.82, w$\kappa$=0.82. Retinoblastoma achieved the highest kappa value (1.0) and lymphoma the lowest (0.66). Of the cases, 16.5% had their original diagnoses amended and the panel confirmed the original diagnosis of paediatric pathologists in 89% of cases compared with 78% for general pathologists. The varying levels of agreement between experts confirm the difficulty of diagnosis in some tumour types, suggesting justification for specialist review in most diagnoses. Specialist training in paediatric pathology is also recommended.

**Keywords:** pathology review; paediatric cancer

Cancer in children is rare, accounting for only 0.5% of all malignancies in all age groups (Stiller, 1992) in developed societies. Treatment is now sophisticated and more specifically targeted at different types and subtypes of disease than in earlier years, but, as it can also have long-term consequences, it is essential that unnecessary effects are avoided by ensuring that the therapy is appropriate to the disease; this demands accurate diagnosis from the outset. The development of new stains and techniques has enhanced diagnostic precision over the years, but there remain cases that are difficult to classify.

The West Midlands Regional Children's Tumour Research Group (WMRCTRG) is a specialist regional registry, holding data on all childhood cancers diagnosed in the West Midlands Health Authority Region (WMHAR) since 1957 (Muir et al, 1992). Histopathological review has been a major element of the Group's work and a large archive collection of slides has been built up. This report describes the analysis of the level of agreement between the reviewers to assess the scale of any variation. We also compared the original diagnoses with those arrived at by the panel. We recognize that in this analysis we are not comparing 'like with like' in that the current reviewers will have had access to stains and techniques that were not available to the original pathologists. However, we chose to undertake this investigation to gain some indication of whether there have been changes in the classification of childhood tumours.

*Received 5 January 1996*
*Revised 6 September 1996*
*Accepted 18 September 1996*

*Correspondence to:* SE Parkes, WMRCTRG, Birmingham Children's Hospital, Ladywood Middleway, Birmingham B16 8ET, UK

### MATERIALS AND METHODS

Cases reviewed comprised all solid tumours diagnosed between 1957–92; leukaemia cases were excluded, because of the poor preservation of bone marrow specimens over time. The review was co-ordinated by Dr A Hugh Cameron (AHC), Consultant Histopathologist at the Children's Hospital, Birmingham (BCH) from 1957–84. Each case was subject to three independent, concurrent opinions i.e., those of AHC and two further pathologists; the 13 referees were recruited on the basis of their professional experience and specialist interest in particular tumour groups.

The review diagnoses were based on at least three sections of the material, one stained with haematoxylin and eosin, this being the major routine diagnostic method (Triche, 1992), and the other

**Table 1** Soft tissue sarcomas: inter-reviewer agreement

| | Referee 1 | | | | | |
|---|---|---|---|---|---|---|
| **Referee 2** | **RMS[a]** | **Other STS** | **Other malignant** | **Benign tumours** | **No tumour** | **Total** |
| RMS | **127** | 1 | – | 1 | – | 129 |
| Other STS | 4 | **38** | 4 | 3 | – | 49 |
| Other malignant | 4 | 3 | **7** | – | – | 14 |
| Benign | 1 | 5 | – | **40** | 1 | 47 |
| No tumour | – | – | – | 3 | **1** | 4 |
| Total | 136 | 47 | 11 | 47 | 2 | 243 |

[a]RMS rhabdomyosarcoma. Percentage of agreement, 88%; kappa statistic, 0.80; weighted kappa, 0.89.

**Table 2** Summary of inter-reviewer analyses, soft tissue sarcoma

| | Percentage agreement | Kappa | Weighted kappa | No. of cases reviewed |
|---|---|---|---|---|
| Ref 1 *vs* Ref 2 | 88 | 0.80 | 0.89 | 243 |
| Ref 1 *vs* Ref 3 | 79 | 0.70 | 0.85 | 96 |
| Ref 2 *vs* Ref 3 | 77 | 0.67 | 0.85 | 91 |
| Overall mean | 81 | 0.72 | 0.86 | |

Ref, referee.

**Table 3** All tumours: inter-reviewer consensus

| Tumour type | Mean percentage agreement | Mean kappa | Mean weighted kappa |
|---|---|---|---|
| Retinoblastoma | 100 | 1.00 | 1.00 |
| Renal tumours | 98 | 0.88 | 0.83 |
| Sympathetic chain | 95 | 0.75 | 0.91 |
| Bone tumours | 94 | 0.89 | 0.79 |
| Germ cell tumours | 93 | 0.87 | 0.77 |
| Hepatic tumours | 90 | 0.83 | 0.71 |
| Epithelial tumours | 89 | 0.85 | 0.63 |
| CNS[a] tumours | 85 | 0.81 | 0.93 |
| Soft tissue sarcoma | 81 | 0.72 | 0.86 |
| Lymphoma | 78 | 0.66 | 0.75 |
| Overall mean | 90 | 0.82 | 0.82 |

[a]CNS tumours, brain and central nervous system.

**Table 4** Cases diagnosed as malignant, deleted after review (23)[a]

| B–M classification of original diagnosis | Original diagnosis | Final diagnosis |
|---|---|---|
| 2a | Hodgkin's disease | Lymphadenitis |
| 2b | NHL | Post-viral syndrome |
| | Anaplastic lymphoma | Inflammation |
| | Malignant NHL | Reactive tissue |
| | Immunoblastic lymphoma | Viral reaction |
| | Reticulum cell sarcoma | Hyperplasia |
| 2e | Histiocytosis X | Reactive tissue |
| 4a | Neuroblastoma | Hamartoma |
| | Neuroblastoma | Malformation |
| 6a | Wilms' tumour | Cystic kidneys |
| 7a | Malignant liver tumour | Cirrhosis |
| 9a | Rhabdomyosarcoma | Benign polyp |
| 11d | Malignant melanoma | Benign melanoma |
| | Malignant melanoma (6) | Naevus |
| | In situ melanoma | Naevus |
| | Basal cell carcinoma | Naevus |
| | Squamous carcinoma | Hyperplasia |
| 11e | Intraductal carcinoma | Hyperplasia |

[a]One case of each, except where specified. Birch – Marsden classification.

two unstained to enable special stains as chosen by the referee if required. No other diagnostic aids were supplied, the pathologists being obliged to treat it as a 'blind' exercise. The review opinions were stored on a computer database and collated when the exercise was completed. When at least two of the three opinions coincided, this was accepted as agreement and confirmed as the final review diagnosis ('consensus').

The Birch–Marsden classification of childhood tumours (Birch and Marsden, 1987) was used, which subdivides the cases into ten major solid tumour groups. Within these, the categories chosen for comparison in the statistical analysis were assigned by three consultant paediatric oncologists (JRM, BJM, MCGS). Distinction was first made between malignant or benign tumour; then opinions were grouped broadly on the basis of differential diagnoses that would involve major treatment variations. Thus, a difference in classification does not merely represent an academic histopathological difference but could have implications for clinical care.

Variability of opinion, even between experts, can be summarized statistically in two ways, the first being a simple measure of unanimity (i.e. the proportion in which there was agreement). However, as this does not take account of the role of chance (i.e. a 'best guess' diagnosis in the event of doubt) or subjectivity in the process, a statistical assessment is desirable. In order to evaluate the consistency between the reviewers, we used the kappa ($\kappa$) statistic (Cohen, 1960, 1968; Maxwell, 1977; Altman, 1990), which is based on a nominal scale of categories for analysis and assesses the agreement between independent observers beyond that which would occur by chance (a value of 1.0 indicates perfect agreement). As interpretation of the kappa statistic is subjective, requiring ad hoc assignment (Bland and Altman 1986; Maclure & Willett 1987, Altman, 1990), we chose the following scale: <0.50, 0.5–0.74, 0.75–0.89 and >0.89, representing poor, fair, good and very good agreement respectively.

Weighted kappa (w$\kappa$) analysis (Cohen, 1968; Altman, 1990) was also included to assess the degree of variation when the opinions differed. This process creates an ordinal scale by assigning graded 'weights' (or penalties) to each category outside the diagonal line that links the agreed cases, according to the number of categories by which it differs. Again, a value of 1.0 denotes no variation between opinions. In this setting, w$\kappa$ demonstrates the clinical significance of the disagreements.

Table 5 All tumours: original *vs* panel diagnoses

| Tumour type | Mean percentage agreement | Mean kappa | Mean weighted kappa |
|---|---|---|---|
| Retinoblastoma | 100 | 1.00 | 1.00 |
| Renal tumours | 96 | 0.68 | 0.69 |
| Germ cell tumours | 93 | 0.86 | 0.77 |
| Sympathetic chain | 92 | 0.62 | 0.71 |
| Bone tumours | 91 | 0.84 | 0.74 |
| Epithelial tumours | 87 | 0.82 | 0.59 |
| CNS tumours | 86 | 0.80 | 0.81 |
| Soft tissue sarcomas | 72 | 0.60 | 0.75 |
| Lymphomas | 68 | 0.54 | 0.71 |
| Hepatic tumours | 65 | 0.37 | 0.50 |
| Overall mean | 85 | 0.71 | 0.73 |

Table 6 All tumours: original *vs* panel by place of diagnosis

| Tumour type | BCH cases (mean) | | | Non-BCH cases (mean) | | |
|---|---|---|---|---|---|---|
| | % | κ | wκ | % | κ | wκ |
| Retinoblastoma | 100 | 1.00 | 1.00 | 100 | 1.00 | 1.00 |
| Bone tumours | 100 | 1.00 | 1.00 | 89 | 0.80 | 0.73 |
| Germ cell tumours | 97 | 0.91 | 0.79 | 96 | 0.91 | 0.77 |
| Sympathetic chain | 97 | 0.83 | 0.87 | 74 | 0.29 | 0.30 |
| Renal tumours | 95 | 0.67 | 0.65 | 97 | 0.49 | 0.28 |
| CNS | 85 | 0.80 | 0.78 | 82 | 0.76 | 0.85 |
| Soft tissue sarcoma | 85 | 0.77 | 0.91 | 56 | 0.42 | 0.47 |
| Hepatic tumours | 78 | 0.58 | 0.64 | 46 | 0.15 | 0.20 |
| Lymphoma | 76 | 0.64 | 0.82 | 63 | 0.49 | 0.57 |
| Epithelial tumours | 73 | 0.62 | 0.39 | 80 | 0.73 | 0.44 |
| Overall mean | 89 | 0.76 | 0.78 | 78 | 0.59 | 0.54 |

### RESULTS

The pathology review began in November 1984 and included cases diagnosed up to the end of 1992. Of the 4592 eligible, 1472 (32%) were leukaemias and were not reviewed. Of the 1116 (24%) brain and CNS tumours, pathology material was reviewed in 482. Of the other 2004 (44%) cases, 382 were not reviewed either because there had not been a biopsy/excision or because the pathology material was no longer available. Thus, 2104 cases underwent review.

The results of the analyses are summarized below, with one category, soft tissue sarcoma (STS), described in detail. Table 1 displays the opinions of two reviewers in the grid formed from the five main categories, the horizontal showing those of the first referee, the vertical those of the second and the diagonal line reveals those cases in which there was agreement (213/243, 88%). The kappa statistic (0.80) shows good agreement between these two reviewers beyond that which could occur randomly and wκ (0.89) implies that when disagreement did occur, the clinical significance of the variation was not major. Table 2 illustrates the results of the review for the whole STS group, in which the large number of disagreements resulted in only fair agreement (κ=0.72), although the high wκ (0.86) suggests that, overall, these disagreements were again not major in terms of clinical significance.

Table 3 summarizes the results for the whole series of tumours using the means of the three separate analyses and shows variations in agreement from 100% in retinoblastoma to 78% in lymphoma. The overall level of agreement was very good (90%), with high κ (0.82) and wκ (0.82) values. Of the ten tumour groupings, only soft tissue sarcoma and lymphoma showed 'fair' agreement (κ<0.75). The effect of wκ on the analyses is seen in CNS tumours which did not have the highest percentage of agreement (85%) but had the highest wκ (0.93) showing that, in the 15% of cases in which disagreements did occur, these were of minor clinical significance. This result could also reflect the number of categories in the analysis, which in turn is influenced by the treatment available for these tumours.

We found that 348 (16.5%) of the 2104 original diagnoses were amended in some way by the review panel. In addition, in 23 cases that had been diagnosed as malignant, the final consensus was that they were not neoplasms, and they were deleted from the Register (Table 4). Table 5 illustrates the results of the comparison between the original and the review diagnoses, in which the difference between the basic percentage of agreement and the kappa values is quite marked. For example, in renal tumours, there was very good agreement (92%) with the original diagnosis, but the relatively low κ (0.68) indicates that the agreement beyond chance was only moderate.

In order to identify any indication of differences between those cases originally diagnosed by paediatric and general pathologists, Table 6 compares the cases originating from BCH (diagnosed by a paediatric pathologist) with those from other hospitals (general pathologists). The panel agreed with the former diagnoses more often than with the latter (κ=0.76 and 0.59 respectively). Only in epithelial tumours (carcinoma) was there better agreement with the panel for those diagnosed outside the paediatric centre (80% *vs* 73%, κ=0.73 *vs* 0.62).

### DISCUSSION

Expert pathology review has been shown to be important in the diagnosis of cancer at any age (Presant et al, 1986; Segelov et al, 1993). For example, Segelov et al (1993), in their review of adult testicular tumours, found that in 28 out of 87 (32%) patients, the diagnosis made on referral to the specialist centre differed from the original. As paediatric tumours are less common and treatment effects potentially more damaging, specialist diagnostic expertise in referral centres is vital. It has been suggested that the results of variation studies of the pathology diagnostic process could and should have an effect on practice (Machin and Parmar, 1994).

The present large study is the first of its kind in paediatric disease, covering the whole spectrum of childhood solid tumours. In addition to comparing initial and final diagnoses, as has often been done in pathology review reports from clinical trials, we have assessed the level of disagreement between reviewers. Freedman and Machin (1993) identified two main issues in the design of observer agreement studies in pathology review, the first being the number and selection of referees. Ours were selected on the basis of their professional experience and specialist interest in specific tumour types, and each case was subject to three opinions, this being deemed appropriate to allow a consensus to be reached. A second requirement is replicate assessment of slides to quantify how much of the observed non-uniformity is due to *intra*-rather than *inter*-observer variability, but this aspect was not assessed in the current study. However, as AHC was both original pathologist for many of the BCH cases and also a member of the panel, we assessed changes in his opinion, as a way of testing for intra-observer differences and found that his diagnosis differed in only 8% of cases (57/664).

© Cancer Research Campaign 1997

The 90% overall level of agreement is good. Kappa and weighted Kappa (0.82 and 0.82 respectively) illustrate further the levels of agreement beyond chance and of variation between reviewers and show that, for most categories, these were also good. This 'league table' of results could be seen as a guideline as to which tumour types might benefit from second opinion before treatment is instituted. It is increasingly common practice for collaborative clinical trials in paediatric oncology to demand specialist review, and our results confirm the justification for this as part of good clinical practice as even small levels of disagreement could have clinical significance for the patient. The degree of inter-observer variability reported underlines the inherent subjectivity and possible limitations of a single diagnosing pathologist in difficult cases. Consistency in terminology and nomenclature is essential and could best be achieved through uniformity of specialist training in paediatric pathology.

The comparison between initial and review diagnoses is limited in retrospective reports by the consideration of advances in knowledge. We have therefore attempted to allow for changes in nomenclature, by grouping 'new' diagnoses under their previous classifications (e.g. primitive neuroectodermal tumour (PNET) with medulloblastoma, etc.). Table 5 shows that, in this comparison, there was less good overall agreement than was seen within the review panel (85% compared with 90%). This illustrates that paediatric pathology has advanced and that tumours can now be more reliably identified (although we are unable to specify further the roles in this study of specialist stains or other factors). Technical advances are continuing to be made, thus constantly improving diagnostic precision, and newer techniques, such as molecular genetics (which is currently more of a research tool), will become indispensable. The analysis by originating hospital shows that paediatric pathologists were more likely to arrive at the panel's definitive diagnosis than were general pathologists. An exception was noted for carcinomas which are generally regarded as 'adult' cancers and which appeared to be more successfully diagnosed by the adult (80% agreement) than the paediatric (73%) pathologists; this presumably reflects their greater familiarity with this form of the disease.

This study shows good overall agreement in the majority of tumour types, although it also demonstrates that, even among experts, identification of clinically significant groupings, based on histopathological examination alone, was not unanimous. This conclusion must be placed in the context that the final diagnosis in the clinical setting does not depend on pathology material alone but is supplemented by other diagnostic information. Our results do not imply, therefore, that any patients will necessarily have been misdiagnosed, and no judgment is implied in the results of this review either of inaccuracy or of infallibility. We do not suggest that there is an association between a change of diagnosis as a result of this review and the appropriateness of original treatment or ultimate outcome. We would simply say that our results support the case for routine review in most childhood tumours to improve the reliability of this component of the diagnostic process.

### ACKNOWLEDGEMENTS

We acknowledge the financial assistance of the Trustees of the Former United Birmingham Hospitals and of the West Midlands Regional Health Authority. We are also very grateful to numerous histopathology departments throughout the region for their co-operation in supplying blocks and sections for review. Our thanks also go to Christina Evans, Darren Redfern and Sue Cavanagh for preparing the slides and to Elvier Forde, Teresa Airey and Noreen Lloyd for their administration of the review procedure.

### REFERENCES

Altman DG (1990) *Practical Statistics for Medical Research*. pp. 403–409 Chapman & Hall: London

Birch JM and Marsden HB (1987) A classification scheme for childhood cancer. *Int J Cancer* **40:** 620–624

Bland D and Altman DG (1986). Statistical methods for assessing agreement between 2 methods of clinical measurement. *Lancet* **1:** 307–310.

Cohen J (1960) A co-efficient of agreement for nominal scales. *Educ Psychol Measurement* **20:** 37–46.

Cohen J (1968) Weighted Kappa: nominal scale agreement with provision for scaled disagreement or partial credit. *Psychol Bull*, **70:** 213–220.

Freedman LS and Machin D (1993) Pathology review in cancer research. *Br J Cancer* **68:** 827–830.

Machin D and Parmar MKB (1994) Pathology review and the diagnosis of cancer. *Lancet* **343:** 55

Maclure M and Willett WC (1987) Misinterpretation and misuse of the kappa statistic. *Am J Epidemiol* 1987; **126:** 161–169

Maxwell AE (1977) Co-efficients of agreement between observers and their interpretation. *Br J Psychiatry* **130:** 79–83.

Muir KR, Parkes SE, Mann JR, Stevens MCG and Cameron AH (1992) Childhood cancer in the West Midlands: incidence and survival, 1980–84, in a multi-ethnic population. *Clin Oncol* **4:** 177–182.

Presant CA, Russell WO, Alexander RW and Fu YS (1986) Soft-tissue and bone sarcoma histopathology peer review: the frequency of disagreement in diagnosis and the need for second pathology opinions. The Southeastern Cancer Group experience. *J Clin Oncol* **4:** 1658–1661

Segelov E, Cox KM, Raghavan D, McNeil E, Lancaster L and Rogers J (1993). The impact of histological review on clinical management of testicular cancer. *Br J Urol* **71:** 736–738

Stiller CA (1992) Aetiology and epidemiology. In *Paediatric Oncology: Clinical Practice and Controversies*, Plowman PN and Pinkerton CR (eds). p. 1. Chapman & Hall London

Triche TJ (1992) Tumour pathology. In *Paediatric Oncology: Clinical Practice and Controversies* Plowman PN and Pinkerton CR (eds). pp. 51–70. Chapman & Hall London

© Cancer Research Campaign 1997